# Prioritization, Incentives, and Resource Use for Sustainable Dentistry: The EU PRUDENT Project

**DOI:** 10.1177/23800844231189485

**Published:** 2023-07-24

**Authors:** S. Listl, O. van Ardenne, J. Grytten, D. Gyrd-Hansen, H. Lang, P. Melo, O. Nemeth, S. Tubert-Jeannin, P. Vassallo, E.B. van Veen, C. Vernazza, R. Waitzberg, J. Winkelmann, N. Woods

**Affiliations:** 1 Radboud University Medical Center, Radboud Institute of Health Sciences (RIHS), Department of Dentistry, Quality and Safety of Oral Healthcare, Nijmegen, Gelderland, the Netherlands; 2 Lygature, Utrecht, the Netherlands; 3 Department of Community Dentistry, University of Oslo, Oslo, Norway; 4 Danish Center for Health Economics, University of Southern Denmark, Odense, Syddanmark, Denmark; 5 Estonian Dental Association, Tallinn, Estonia; 6 Instituto de Saúde Pública da Universidade do Porto, Porto, Portugal; 7 Department of Community Dentistry, Semmelweis University, Budapest, Hungary; 8 University of Clermont-Auvergne, UFR d’Odontologie, Clermont-Ferrand, France; 9 Ministry for Health, Health Promotion and Disease Prevention Directorate, Valetta, Malta; 10 Newcastle University, School of Dental Sciences, Newcastle, Tyne and Wear, UK; 11 Department of Health Care Management, Technische Universität Berlin, Faculty of Economics & Management, Berlin, Germany; 12 European Observatory on Health Systems and Policies, Brussels, Belgium; 13 University College Cork, Cork University Business School, Centre for Policy Studies, Cork, Ireland

**Keywords:** health finance, oral health, health policy, workforce, stakeholder participation, citizens

## Abstract

PRUDENT (Prioritization, incentives and Resource use for sUstainable DENTistry) is a multinational project funded under the European Union’s (EU’s) Horizon Europe program. Our team includes partners from Denmark, Estonia, France, Germany, Hungary, Ireland, Malta, the Netherlands, Norway, Portugal, and the United Kingdom; we aim to develop and implement an innovative and context-adaptive framework for optimal financing of oral health care that enables access to essential oral health care for everyone without causing financial hardship. PRUDENT will leverage health economics and implementation science methods to convert novel evidence on oral health financing into meaningful improvements of oral care. PRUDENT will harness behavioral experiments, system dynamics modeling, implementation trials, deliberative processes, and a multicountry monitoring framework on oral health care financing in the EU. Using a mixed-methods research design, PRUDENT addresses 3 objectives: (1) to develop a harmonized core set of oral health system indicators, implement them in a novel EU-wide monitoring system, and integrate them in deliberative processes to set priorities for oral care financing; (2) to identify optimization strategies for oral health care financing, in which real-world and lab experiments on provider payment and oral care insurance coverage, needs-adaptive resource planning, regulatory learning, and digital decision aid tools are leveraged to help accelerate transformations in oral care financing; and (3) to harness innovative knowledge transfer strategies for the co-development and co-production of sustainable implementation strategies for oral and general health care financing. Through improving access to essential oral care for everyone without causing financial hardship, PRUDENT is expected to help achieve universal health coverage for oral health.

**Knowledge Transfer Statement:**

The EU PRUDENT project aims to enhance the financing of oral health systems through novel evidence and implementation of better financing solutions together with citizens, patients, providers, and policy makers. The multicountry nature of the project offers unique windows of opportunity for rapid learning and improving within and across various contexts. PRUDENT is anticipated to strengthen capacities for better oral care financing in the EU and worldwide.

## Why PRUDENT?

The 2021 World Health Organization (WHO) Oral Health Resolution highlighted that oral health has been a dramatically neglected area in health policy for many years (WHO 2021). This also has repercussions for the financing of oral health care, which is often separated from the general health system and differs considerably across countries. Enormous cross-country differences exist with respect to the raising of revenues, remunerating providers, incentivizing consumers, workforce planning, and the purchasing of oral health care services and products.

There are major challenges in the financing of oral health care: while oral diseases are the third most expensive diseases to treat in the European Union (EU), many citizens cannot afford to access essential oral health care (Listl et al. 2019; WHO Europe 2019). Oral diseases are a main driver of catastrophic health expenditures and particularly affect poorer and marginalized groups; this causes detrimental impacts for the individual citizen while increasing costs and wasting resources on the macro level (Listl et al. 2021; WHO 2022). The currently predominant provider payment system (fee-for-service) has been associated with supplier-induced demand, and existing methods to plan the oral health workforce are not sufficiently responsive to people’s oral care needs (Listl et al. 2019). Previous attempts to reform the financing of oral care have revealed major implementation challenges due to misaligned stakeholder interests and a culture of reluctance to change.

Solving such complex problems as described above requires a comprehensive deciphering of oral care–financing mechanisms and the development and implementation of context-specific improvement strategies together with citizens/patients, service providers, payers, and policy makers.

## PRUDENT Aims

**Figure 1.**
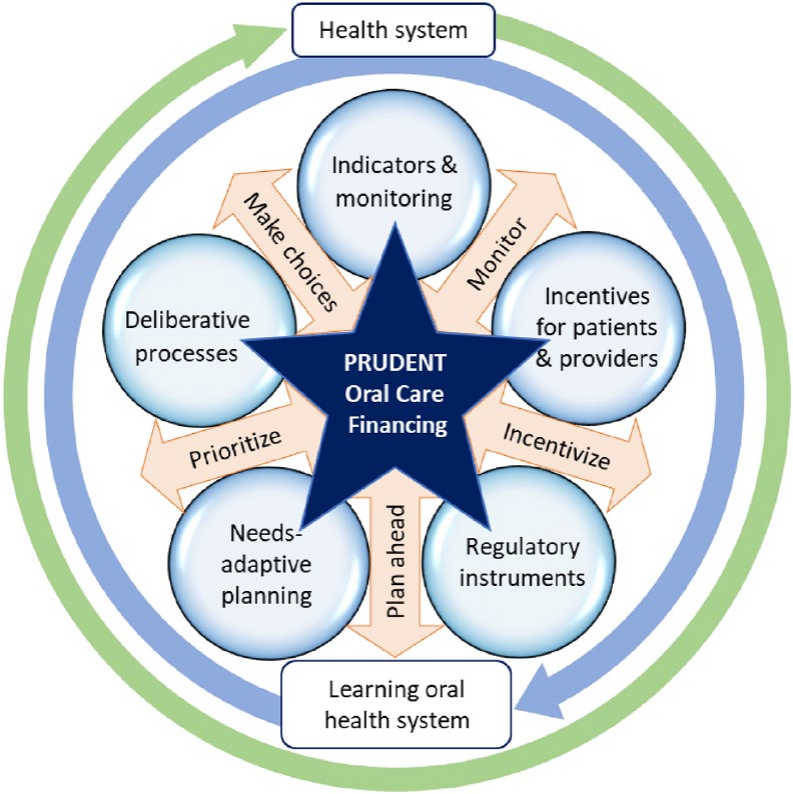
The PRUDENT financing model will provide a blueprint model for co-developing better financing models for oral and general health systems.

The PRUDENT project aims to innovate the financing of oral health systems through novel evidence and implementation of better financing solutions together with citizens, patients, providers, and policy makers. The key challenge is to create a learning oral health system that converts indicators, provider and patient incentives, regulatory instruments, needs-adaptive planning, and deliberative processes into sustainable health improvements. The ultimate deliverable will be the PRUDENT financing model (see Fig. 1), implemented in the form of support tools to implement better oral financing, which will be combined within the PRUDENT Financing Companion (see below).

The specific objectives of the PRUDENT project are:

- **Objective 1:** To develop a harmonized core set of oral health system indicators, implement them in a novel EU-wide monitoring system, and integrate them in deliberative processes to set priorities for oral care financing;
- **Objective 2:** To identify optimization strategies for oral health care financing. Real-world and lab experiments on provider payment and oral care insurance coverage, needs-adaptive resource planning, regulatory learning, and digital decision aid tools will be leveraged to help accelerate transformations in oral care financing.
- **Objective 3:** To harness innovative knowledge transfer strategies for the co-development and co-production of sustainable implementation strategies for oral health care financing.

## PRUDENT Methods

**Figure 2.**
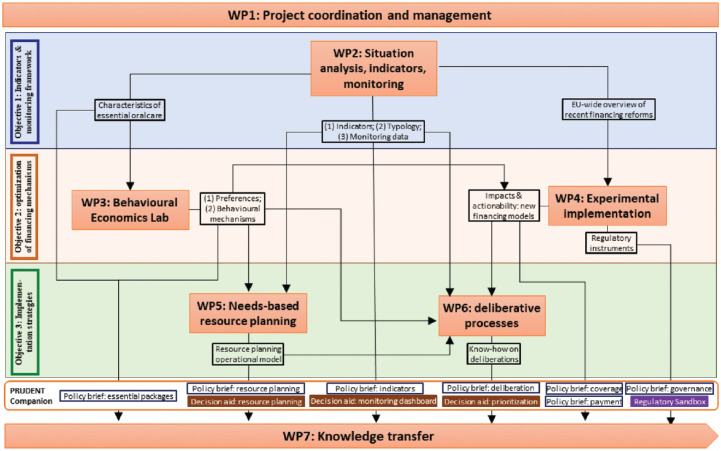
PRUDENT’s Work Packages (WPs) and how they interrelate.

PRUDENT builds on state-of-the-art health economics, health systems, and implementation science methods. In particular, PRUDENT leverages the following methods, concepts, and models (also see Fig. 2):

### Objective 1: Oral Health System Indicators and EU-Wide Monitoring Framework

- **Situational analysis:** PRUDENT will comprehensively map the current status of oral health financing in European countries and use this new knowledge to develop a typology of oral health financing in Europe. The situational analysis will include a scoping review and an expert survey, which will feed into the typology of financing and payment systems in oral health care. The findings will help provide understanding of the context for improving oral care financing in subsequent PRUDENT steps.
- **Consenting of candidate items for essential oral health care benefit baskets:** A scoping review will identify a preliminary list of previously suggested essential items of oral health care. Structured group brainstorming will then serve to critically reflect on this preliminary list of items, reach a collective understanding of what constitutes essential oral health care, and establish a refined list of consented candidate items for essential oral health care benefit baskets. These candidate items for essential oral health care are envisioned to inform future priority setting.
- **Deliberative processes toward implementation of cost-effective and equitable oral health coverage:** Priority setting and resource allocation (PSRA) processes have gained increasing traction in addressing health care resource allocation problems given their ability to incorporate multiple stakeholders, address multiple competing objectives, incorporate evidence, and provide transparent, pragmatic, and flexible decision making. PRUDENT will undertake case studies in select countries to test the applicability of PSRA in relation to oral health coverage decision.
- **Co-production of a harmonized set of oral health system performance indicators:** Guided by the WHO Health Systems Framework, PRUDENT will initially map existing oral health system performance indicators with a specific focus on financing aspects. Based on the list of identified indicators, a modified RAND/UCLA appropriateness method will be applied to consent a multicountry harmonized set of oral health system performance indicators. The indicators will form the basis to operationalize PRUDENT’s monitoring framework.
- **EU-wide monitoring framework for oral health systems performance:** Based on the identified performance indicators and essential items for oral care (see above), PRUDENT will develop a multicountry monitoring framework for oral health systems performance. Available data sources with relevant oral health system information will be mapped. PRUDENT will also carry out an online survey of EU citizens’ perception of oral health systems performance in select countries. The information from the citizen survey and existing data sources will be incorporated in a cross-country interactive dashboard.

### Objective 2: In-Depth Analysis of Optimization Strategies for Oral Care Financing

- **Behavioral economics lab to decipher the impacts of incentives on providers’ and citizens’ choices:** To improve the understanding of demand- and supply-side behavioral mechanisms in the context of oral care markets, PRUDENT builds on state-of the-art stated preference methods to generate an in-depth understanding of how providers/citizens respond to incentives and infrastructures of oral care markets. Discrete choice experiments will serve to identify citizens’ preferences for different dimensions of oral health (particularly oral health–related quality of life), the impact of potential barriers to demand (e.g., out-of-pocket payments), and personal characteristics such as risk aversion and time preferences. Provider preferences will be elicited to decipher their extrinsic and intrinsic motives. In addition, linkage of data from the discrete choice experiments with registry data from Denmark will allow comparisons of real-life choices with stated preferences.
- **Practice-based study on SES–risk-adjusted capitation payment in the Netherlands** (SES = Socio-Economic Status): Leveraging the experimentation clause of the Dutch Health Care Authority (NZA) and a committed stakeholder ecosystem comprising research organizations, health insurers, oral health professionals, and patients, PRUDENT will carry out a practice-based study in the Netherlands to test the implementation of SES–risk-adjusted capitation payments (high SES vs. low SES). The new payment system (= intervention) aims to reduce social inequalities in children/adolescent oral care. To compare the intervention group with the current standard of care (= fee for service), a synthetic control group will be constructed based on routine health insurance data. The evaluation will comprise an outcome evaluation, a process evaluation, and an economic evaluation. Given the dearth of empirical evidence on implementing new payments systems, this practice-based study provides a highly unique opportunity to evaluate the impact and decipher the facilitators and barriers involved in payment systems transformation.
- **Analysis of a population-wide reform to achieve UHC for oral care in France:** Recent reforms in France (i.e., the “100% Santé” scheme), have aimed to improve access to dental care, especially for lower-income groups. These reforms emerged in response to “participative democracy” processes during the 2017 French presidential campaign. The resulting program has started offering an array of prosthetics, ranging from removable to fixed prostheses and crowns/bridges, the cost of which is now being reimbursed by compulsory health insurance. PRUDENT will harness large-scale administrative data to evaluate the extent to which the French “100% Santé” scheme has been achieving UHC for oral care. For methodological triangulation, relevant stakeholders and experts will be interviewed to provide additional insights in and perspectives on the “100% Santé“ scheme.
- **Multicountry contextualization of oral care–financing reforms**: To further enhance the knowledge about the impacts and implementation of oral care–financing reforms, PRUDENT will carry out a realist review to unpack the configurative elements of financing reforms/arrangements in various European countries. This will serve to develop a more particularized understanding of the context-specific adaptability and potential impacts of alternative oral care–financing arrangements.
- **Needs-adaptive resource and workforce planning for oral health care:** The rigidity of current resource- and workforce-planning models is a barrier for potential efficiency gains. To this end, PRUDENT will systematically identify the extent and impacts of dynamic changes in population oral care needs (e.g., due to changes in oral health morbidity or emergence of new oral care delivery approaches). A system dynamics model will be developed to explicitly take account of multiple factors that influence peoples’ oral care needs and to be applicable in the context of the participating European countries.
- **Regulatory learning to improve oral care financing:** Oral care–financing arrangements are governed by EU-wide and county-specific regulatory frameworks. PRUDENT will systematically map currently existing regulatory frameworks for oral care financing and their potential for positive change. The identified regulations for oral care financing will feed into the “PRUDENT Regulatory Sandbox” to support policy and decision makers.

### Objective 3: Co-develop and Co-produce Sustainable Implementation Strategies

- **PRUDENT Financing Companion:** To reflect on project results with policy makers, citizens/patients, providers, and researchers, a participatory conference will be organized. The feedback received will be consolidated as implementation support tools: (1) policy briefs for national and EU policy makers, (2) decision aids (e.g., for oral health systems monitoring, workforce planning, priority setting), and (3) regulatory sandbox (inventory of regulations through which oral care financing can be improved).
- **European observatory policy workshop:** In collaboration with the European Observatory on Health Systems and Policies, this workshop will bring together key findings from PRUDENT and international experts from policy and practice from various countries. The policy workshop will be geared toward knowledge brokering of evidence and insights into policy makers’ views on concrete implementation.
- **Executive leadership module:** A training module on oral health financing will be developed for policy makers from all relevant health sectors (also beyond oral health).

## PRUDENT’s Expected Results and Impacts

PRUDENT’s key results are expected to be provided via the PRUDENT Financing Companion:

- Core set of indicators for oral care systems performance monitoring
- Know-how for tailoring essential packages of oral care and insurance coverage to citizen preferences
- Know-how for designing incentives according to provider behaviors
- Know-how for implementation of oral care financing reforms and innovations
- Know-how for needs-adaptive oral health resource and workforce planning
- Know how for leveraging deliberative processes for better priority setting
- Implementation support tools: (1) policy briefs, (2) decision aids, and (3) regulatory sandbox

In the longer run, successful operationalization of the PRUDENT Financing Companion is expected to scale up social capacity for optimization of oral care financing in the EU. Improved financing arrangements are expected to make oral health care more effective, efficient, accessible, resilient, trusted, and sustainable, both fiscally and environmentally. It is anticipated that citizens will benefit from improved access to oral health services, including financial risk protection and timely access to essential oral health services; oral care providers will save resources by using innovative technologies and reorganizing workflows; health policy and systems will adopt a holistic approach for the evaluation of oral health outcomes, the value of oral health interventions, the organization of oral care, and all related decision making. Ultimately, PRUDENT is expected to enhance the integration of oral health in general health systems, thereby improving the access to innovative, sustainable, and high-quality oral and general health care.

## Conclusions

The EU PRUDENT project addresses several “know-how” and “know-do” gaps to achieve a much-needed step change in improving the financing of oral health systems. In the sense of “learning oral health systems,” the multicountry nature of the project offers unique windows of opportunity for rapid “learning and improving” within and across various contexts. Eventually, PRUDENT can strengthen capacities for better oral care financing in the EU and worldwide, thereby contributing to WHO’s Universal Health Coverage goal “that all individuals and communities have access to essential, quality health services that respond to their needs and that they can use without suffering financial hardship” (WHO 2022).

## Author Contributions

S. Listl contributed to conception, design, drafted and critically revised the manuscript; O. van Ardenne, J. Grytten, D. Gyrd-Hansen, H. Lang, P. Melo, O. Nemeth, S. Tubert-Jeannin, P. Vassallo, E.B. van Veen, C. Vernazza, R. Waitzberg, J. Winkelmann, N. Woods contributed to conception, design, critically revised the manuscript. All authors gave final approval and agree to be accountable for all aspects of the work.
